# A15 LEVERAGING CULTURE-DEPENDENT AND -INDEPENDENT METAGENOMICS TO IDENTIFY ENTEROBACTERIACEAE GENES ASSOCIATED WITH ACTIVE ULCERATIVE COLITIS

**DOI:** 10.1093/jcag/gwad061.015

**Published:** 2024-02-14

**Authors:** D Tertigas, F Rinawi, P Moayyedi, A Griffiths, M Surette

**Affiliations:** McMaster University Faculty of Health Sciences, Hamilton, ON, Canada; Technion Israel Institute of Technology The Ruth and Bruce Rappaport Faculty of Medicine, Haifa, Haifa, Israel; McMaster University Faculty of Health Sciences, Hamilton, ON, Canada; The Hospital for Sick Children, Toronto, ON, Canada; McMaster University Faculty of Health Sciences, Hamilton, ON, Canada

## Abstract

**Background:**

Ulcerative colitis (UC) is a type of inflammatory bowel disease (IBD) that is restricted to the large intestine and is characterized by mucosal inflammation. There is evidence that pathogenic bacteria contribute to UC pathogenesis in at least some patients and the bacterial family Enterobacteriaceae are prime suspects. Some strains of Enterobacteriaceae carry virulence genes important for colonizing the gut (e.g. *fimH*) and disrupting the intestinal epithelium (e.g. hemolysins). We hypothesize that key virulence genes carried by strains of many Enterobacteriaceae species contribute to disease activity in some UC patients. As we predict virulence genes are strain-specific, the detection of the species alone (e.g. *Escherichia coli*) is insufficient to predict pathogenic potential and this has confounded previous studies to identify infectious agents in UC.

**Aims:**

We aim to use culture-dependent and -independent sequencing approaches to identify Enterobacteriaceae genes in the UC gut microbiome associated with active disease.

**Methods:**

UC patient stool samples collected throughout enrolment in randomized control trials of fecal microbiota transplantation (FMT) for adult UC (n=10) and microbiome studies in early-onset pediatric UC (n=25) were cultured on MacConkey (MAC) agar to enrich for Enterobacteriaceae. Strains were isolated from baseline stool samples cultured on MAC agar for whole genome sequencing and virulence gene characterization. Baseline and post-treatment stool samples cultured on MAC agar were sent for metagenomic sequencing. Using a subset of 11 pediatric patients, I developed a metagenomic pipeline to identify Enterobacteriaceae genes enriched during active UC compared to periods of remission or milder disease.

**Results:**

Known virulence genes, including *fimH* and hemolysins, were present in some strains across multiple Enterobacteriaceae species. Using an unbiased metagenomic approach with the pediatric cohort, we identified 42 genes enriched at baseline with the criteria they must be elevated in at least three of the 11 patients. The majority of the 42 genes were distributed into six gene clusters, including a small plasmid.

**Conclusions:**

Our approach allows us to identify genes enriched in active UC that have not been previously described in the literature and our analysis will be repeated with our adult cohort. Genes that are enriched in active UC in the pediatric and/or adult cohort will be validated in publicly available metagenomic datasets that consist of both IBD patients and healthy controls. Furthermore, culture-dependent approaches allow us to test mechanisms in vivo that are informed by our bioinformatics. Leveraging microbiome and clinical data provides a unique window to guide future diagnostics and treatments to improve outcomes for UC patients.

**Funding Agencies:**

CIHROGS

